# Assessment of persistent antimicrobial and anti-biofilm activity of p-HEMA hydrogel loaded with rifampicin and cefixime

**DOI:** 10.1038/s41598-022-07953-3

**Published:** 2022-03-10

**Authors:** Ola Tarawneh, Hadeel Abu Mahfouz, Lama Hamadneh, Ahmad A. Deeb, Iyad Al-Sheikh, Wasan Alwahsh, Abdulqader Fadhil Abed

**Affiliations:** Department of Pharmacy, Faculty of Pharmacy, Al-Zaytoonah University of Jordan, P. O. Box 130, Amman, 11733 Jordan

**Keywords:** Microbiology, Materials science

## Abstract

Catheter-associated urinary tract infections (CAUTIs) are nosocomial infections causing more than one million hospital cases annually. The progress of CAUTIs leads to severe health complications. Infections result in blockage of the medical device due to biofilm formation, which necessitates the replacement of the device. The objective of this study is to improve urological biomaterials to minimize microbial growth and reduce the incidence of CAUTIs. Challenges from mixed biofilm are crucial and need to be addressed in the development of new coating materials. Herein, an investigation highlighted the reduction of mixed biofilm overgrowth and attachment tendency on poly-2-hydroxyethyl methacrylate (p-HEMA) surface by loading the hydrogel with rifampicin (RIF), cefixime trihydrate (CFX), and combined ratios of RIF and CFX. Mixed biofilm-formation ability in (3:1) RIF: CFX-loading p-HEMA (F6) surface showed best tendency to resist form biofilm. Persistent antimicrobial activity increased in p-HEMA loaded with combined ratios of RIF and CFX surface compared to p-HEMA alone, antimicrobial activity lasted for 8 days. All fabricated films exhibited %cell viability higher than 75% on HEK 293 cells. The addition of RIF and CFX may improve the duration of urological device employment before replacement.

## Introduction

**Figure 1:**
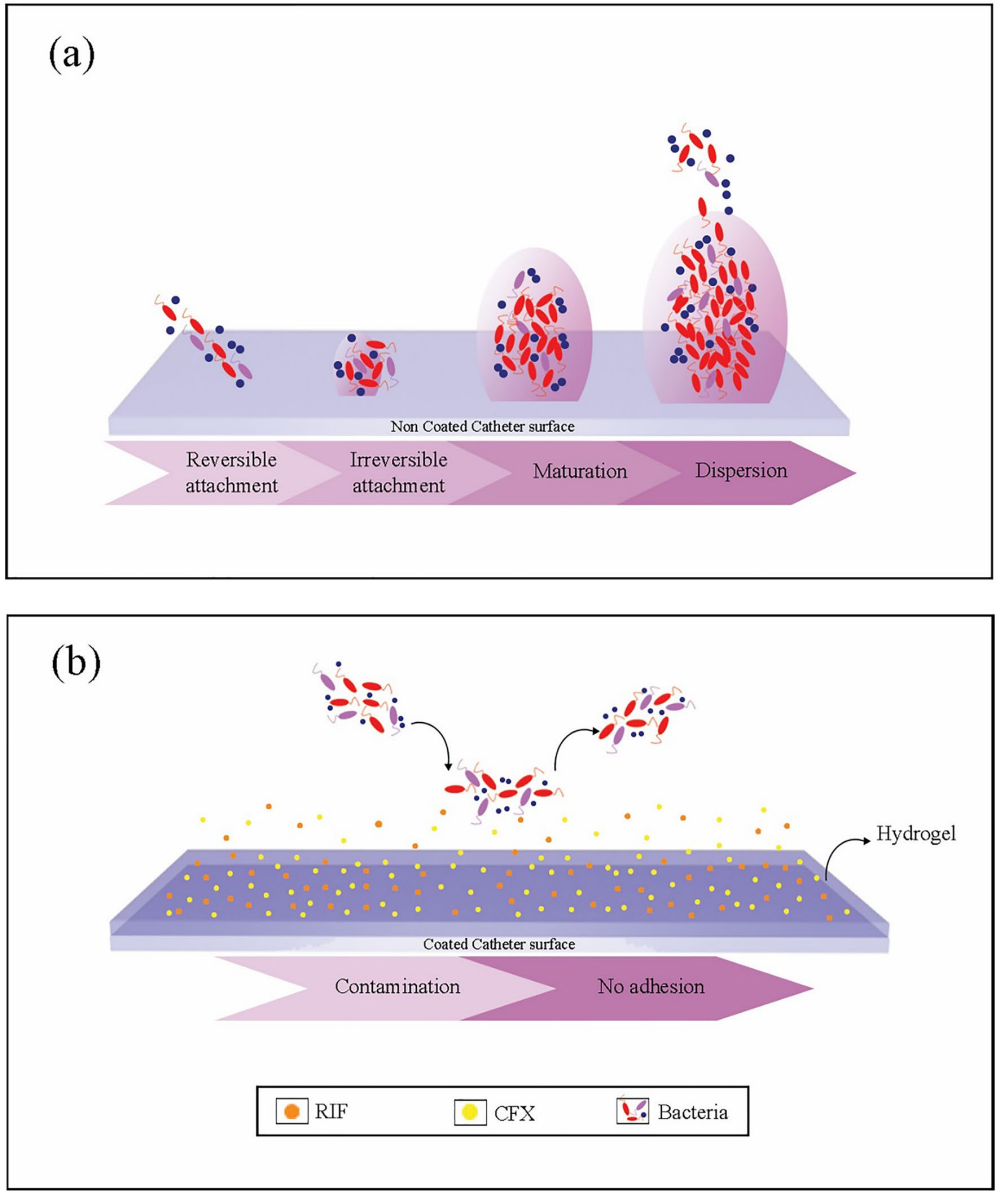
(**a**) Biofilm formation steps on non-coated surface showing reversible attachment of different strains of bacteria, irreversible attachment of bacteria, maturation of small cluster, maturation of large cluster and dispersion of bacteria. (**b**) The attachment of different strains of bacteria on coated surface where bacteria do not attach to the surface and thus do not have opportunity to biofilm formation.

Catheter-associated urinary tract infection (CAUTI) accounts for 40% of hospital-acquired infections (HAIs)^1^. The presence of 100,000 CFU/mL (colony-forming unit per 1 mL) in urine culture requires therapeutic interference. During catheterization, the presence of/or greater than 100 CFU/mL necessitates immediate therapeutic interference^2,3^^.^ CAUTI is caused by the migration of microorganisms from the skin enclosing the urethral opening into the urinary tract or by the catheter itself if an aseptic technique is not applied^4^. Microorganisms connect to the formed adhered protein layer and attach to the surface^5^. The adhesion of bacteria on the catheter surface is governed by reversible weak forces such as Van der Waals or electrostatic attraction forces and then by the formation of irreversible strong forces like covalent bonds^6^. Bacteria attached to the biomaterial surface leads to subsequent colonization and eventually provoke the formation of biofilms^7–9^ as illustrated in Fig. 1.

Biofilms mainly comprise multiple species community often referred to as mixed biofilms^10,11^. The rate of persistent infections caused by mixed biofilms are higher than infections caused by mono-species^12,13^. Mixed biofilms increase the incidence of bacteriuria, encrustation, catheter blockage, and microbial resistance to antibiotic^14,15^. Those complications raise serious critical health problems such as acute pyelonephritis, prostatitis in males, cystitis, urinary stones, renal failure, and even death^15,16^. To date, the only way to treat an infected urinary catheter is by removing it and insert a new device which increases the suffering of patients, increases the burden on medical staff as well as the economic cost^7,17^.

Classical urinary biomaterials were made from latex, but those were limited due to high levels of infection rate and allergy complains^4,18^. Then, several biomaterials were involved in the manufacture of urinary devices such as silicone, polyurethane where every material provided improvement. However, all materials demonstrated infection and biofilm formation^19^. Another approach proposed was coating the medical device with biomaterials documented to be chemically stable, economical, and patient-friendly, in addition to their crucial ability to reduce infection and prevent biofilm-formation^20^. The improvement of the coating layer occurred by using hydrophilic coatings^21^, pH-responsive coatings^22^, antifouling coatings^23^, and poly-zwitterion coatings^24^, coating layer by silver^25^, nitric oxide^26^, microbicides^27^, and antimicrobial peptides^28^.

Microbicides-coated catheters were a strategy adopted to inhibit or delay the onset of CAUTIs^29^. Rifampicin (RIF), and cefixime trihydrate (CFX) are effective against Gram-positive and Gram-negative pathogens with antibiofilm properties^30–32^. CFX is effective in the treatment of a broad variety of bacterial infections, such as urinary tract infection^32^.

The use of polymeric coating comprises approaches dictated by the physicochemical properties of the polymer. For example, pH-sensitive polymers are polyelectrolytes that have ionizable function groups in their structure that either lose or accept protons and dissociate in response to differences in environmental pH^33^. poly-2-hydroxyethyl methacrylate) (p-HEMA) is a pH-sensitive polymer that contains primary alcohol^34^, with hydrophilic and lipophilic balance, non-toxic, transparent, insoluble^35^, can’t be absorbed by the body^36^, resistant to degradation^37^, has stable mechanical properties^38^. p-HEMA was used in contact lenses, wound dressings, controlled drug release delivery systems, and implants^39^.

The aim of this study was to develop and characterize urological biomaterials fabricated from 2-hydroxyethyl methacrylate (2-HEMA) and loaded with RIF, CFX, and combined ratios. Moreover, we report for the first time the assessment of anti-fouling ability of RIF/CFX hydrogel against mixed biofilms formation.

## Results

In this study, p-HEMA was loaded with RIF, CFX, and combined ratios of RIF and CFX. Characterization of the fabricated films takes place to determine T_*g*_, U.T.S, elongation%, and Young’s modulus. Study the EWC% and evaluate HEK 293 cells cytotoxicity. Various types of bacteria were used to determine the persistence antimicrobial activity for 8 days and biofilm-formation ability.

### Thermal analysis using dynamic mechanical thermal analyser (DMTA)

**Figure 2:**
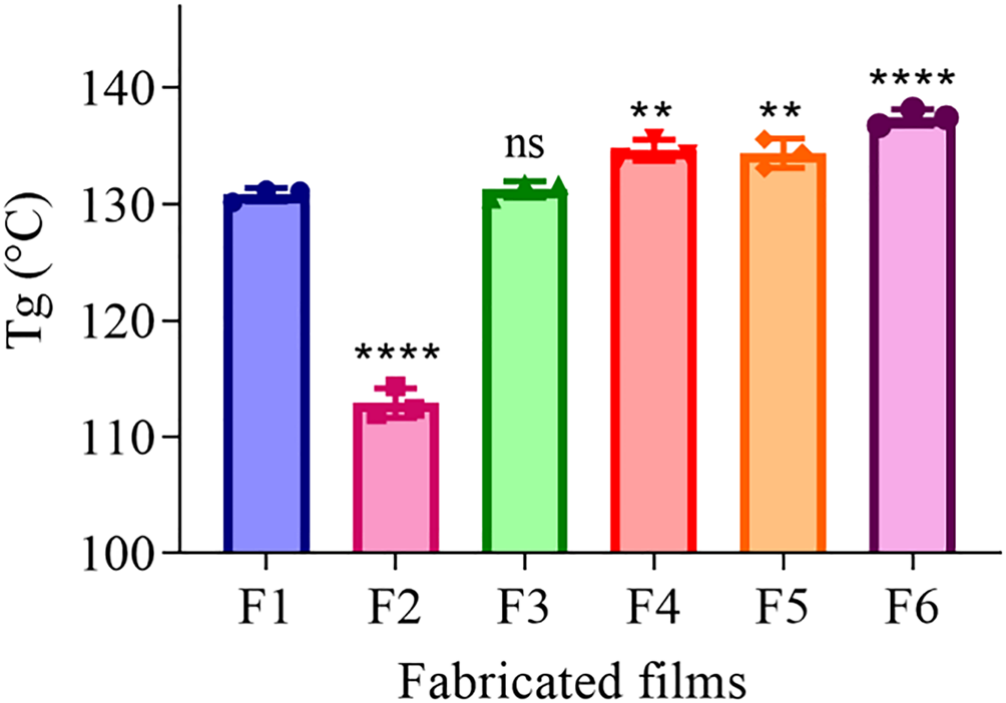
The T_*g*_ values of the dried films of p-HEMA and drugs-loaded p-HEMA, where (F1): p-HEMA, (F2): RIF-loaded p-HEMA, (F3): CFX-loaded p-HEMA, (F4): (1:1) RIF:CFX-loadedp-HEMA, (F5): (1:3) RIF:CFX-loaded p-HEMA, (F6): (3:1) RIF:CFX-loaded p-HEMA. Data are obtained form DMTA and presented as mean ± SD, (*n* = 3). **p-value < 0.01, ****p-value < 0.0001 significant difference between fabricated films.

As presented in Fig. 2, p-HEMA (F1) had a T_*g*_ of 130.82 ± 0.57 °C. The employment of microbicide showed an effect on T_*g*_, for instance, loading p-HEMA with RIF in a ratio of 0.5% (*w/w*) (F2) decreased the T_*g*_ to 112.89 ± 1.27 °C (****p-value ≤ 0.0001). Loading p-HEMA with CFX (F3) showed similar T_*g*_ (131.28 ± 0.69 °C, *p* = 0.98) compared to p-HEMA. On the other hand, the *T*_*g*_ for microbicide combined ratios that are loaded in p-HEMA (F4, F5, and F6) the observed T_*g*_ was higher than control 134.64 ± 0.89 °C (**p-value ≤ 0.0024), 134.36 ± 1.26 °C (**p-value ≤ 0.0044), and 137.47 ± 0.70 °C (****p-value < 0.0001) respectively.

### Mechanical analysis

**Figure 3:**
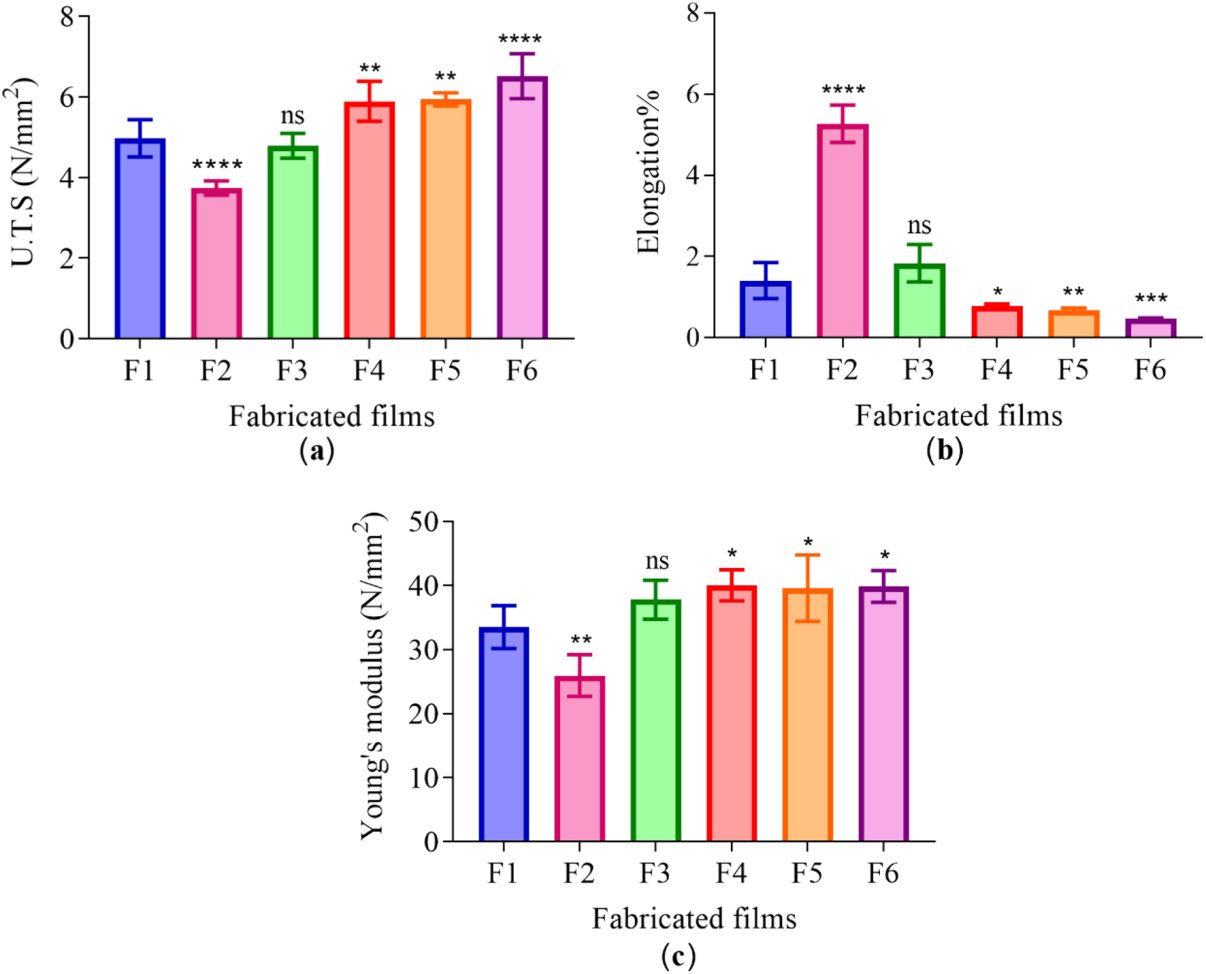
Mechanical properties of the dried films, where (**a**) U.T.S, (**b**) elongation%, and (**c**) Young’s modulus. (F1: p-HEMA, F2: RIF-loaded p-HEMA, F3: CFX-loaded p-HEMA, F4: (1:1) RIF:CFX-loadedp-HEMA, F5: (1:3) RIF:CFX-loaded p-HEMA, F6: (3:1) RIF:CFX-loaded p-HEMA). Data are presented as mean ± SD, (*n* = 3). *p-value < 0.05, **p-value < 0.01, ***p-value < 0.001, ****p-value < 0.0001 significant difference between fabricated films.

The influence of drug type and ratio on the tensile strength, elongation%, and Young’s modulus of the dried films was studied. The U.T.S of p-HEMA (F1) was found to be 4.97 ± 0.46 N/mm^2^. The addition of 0.5% (*w/w*) RIF into p-HEMA (F2) decreased the U.T.S to 3.74 ± 0.18 N/mm^2^ (****p-value < 0.0001). Loading p-HEMA with CFX (F3) alone did not affect U.T.S and it was similar to F1. Employing both microbicides in different ratios of CFX and RIF increased the U.T.S significantly compared to F1. The U.T.S of F4, F5, and F6 increased to 5.89 ± 0.50 N/mm^2^ (**p-value = 0.004), 5.94 ± 0.17 N/mm^2^ (**p-value = 0.002), and 6.51 ± 0.56 N/mm^2^ (****p-value < 0.0001) respectively, as presented in Fig. 3a.

As for elongation% of p-HEMA (F1) was 1.41 ± 0.44%. RIF-loaded p-HEMA made the elongation% increase five time to 5.27 ± 0.46% (****p-value < 0.0001). The elongation% of CFX-loading p-HEMA (F3) was similar to F1 (1.83 ± 0.46%, *p*-value > 0.05). Furthermore, employing combined ratios of RIF and CFX decrease the elongation% to values ranging from 0.47% to 0.78% (*p-value < 0.05), as shown in Fig. 3b.

Young’s modulus, it was 33.51 ± 3.36 N/mm^2^ for p-HEMA (F1). Whereas RIF-loaded p-HEMA (F2) decreased the modulus to 25.94 ± 3.25 N/mm^2^ (**p-value = 0.007). CFX-loaded p-HEM (F3) did not affect the elastic modulus of p-HEMA significantly. However, employing combined ratios of CFX and RIF increased the modulus significantly, as presented in Fig. 3c.

### Equilibrium water content (swelling)

**Figure 4:**
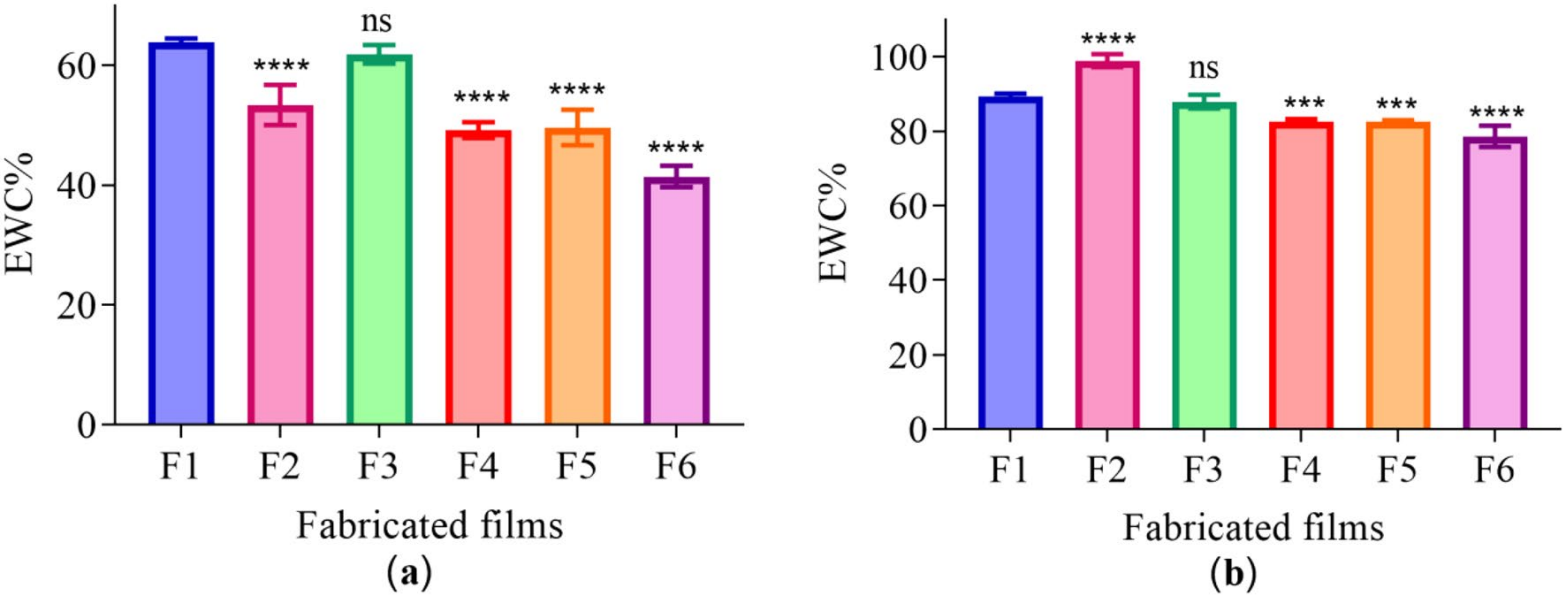
EWC% for dried films after 24 h at 20 °C (**a**) pH 5 and (**b**) pH 9. Data are represented as mean ± SD, (*n* = 6). ***p-value < 0.001, ****p-value < 0.0001 significant difference between fabricated films.

As presented in Fig. 4a,b. The EWC% at pH 5 for fabricated films loaded with drugs decreased in comparison to p-HEMA (F1). Whereas the EWC% at pH 9 for RIF-loaded p-HEMA increased in comparison to p-HEMA (F1) (98.97 ± 1.74%) (****p-value < 0.0001). Loading p-HEMA with a combined ratios of RIF and CFX decreased the EWC% in (F4, F5, and F6) to 82.67 ± 0.67% (***p-value = 0.0002), 82.56 ± 0.40% (***p-value = 0.0002), and 78.64 ± 2.84% (****p-value < 0.0001) respectively. Furthermore, no marked change was observed in CFX loaded *p-*HEMA (F3).

### Cytotoxicity evaluation of the fabricated hydrogel using (MTT assay)

**Figure 5:**
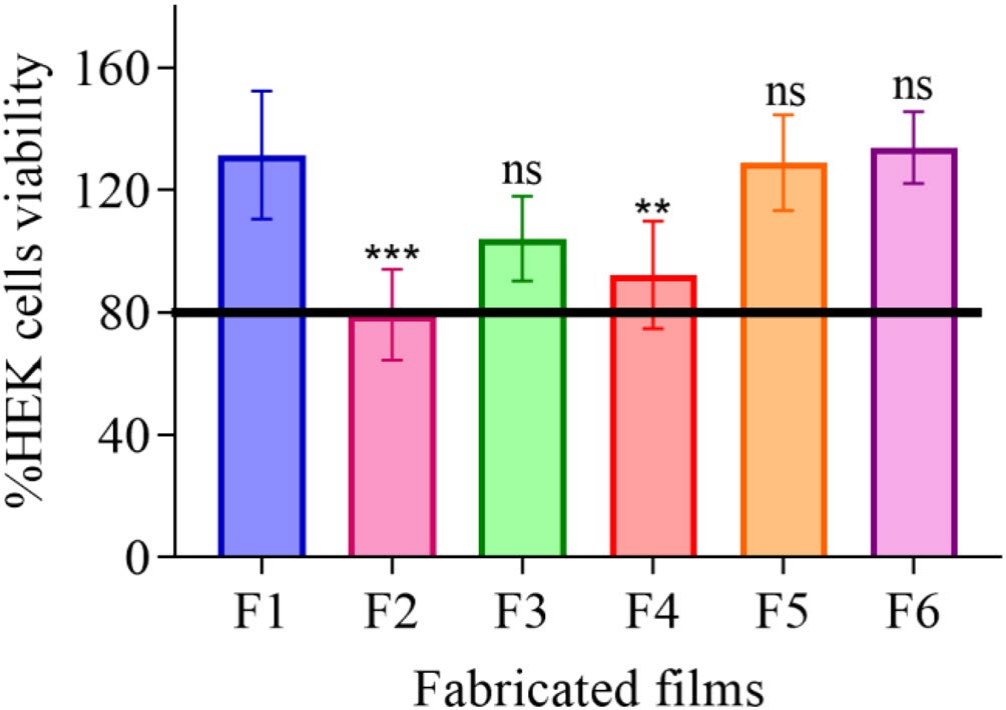
The %cell viability for the dried films. Where (F1): p-HEMA, (F2): RIF-loaded p-HEMA, (F3): CFX-loaded p-HEMA, (F4): (1:1) RIF:CFX-loadedp-HEMA, (F5): (1:3) RIF:CFX-loadedp-HEMA, (F6): (3:1) RIF:CFX-loadedp-HEMA. %. Data are presented as mean ± SD, (*n* = 6). **p-value < 0.01, ***p-value < 0.001 significant difference between fabricated films.

The %cell viability of p-HEMA control (F1) was 131.43 ± 20.96. The %cell viability for RIF-loaded p-HEMA (F2) was lower than 80%. In addition, the %cell viability for F3, F4, F5, and F6 was higher than 90%, as shown in Fig. 5.

### Persistence of antimicrobial activity

**Figure 6:**
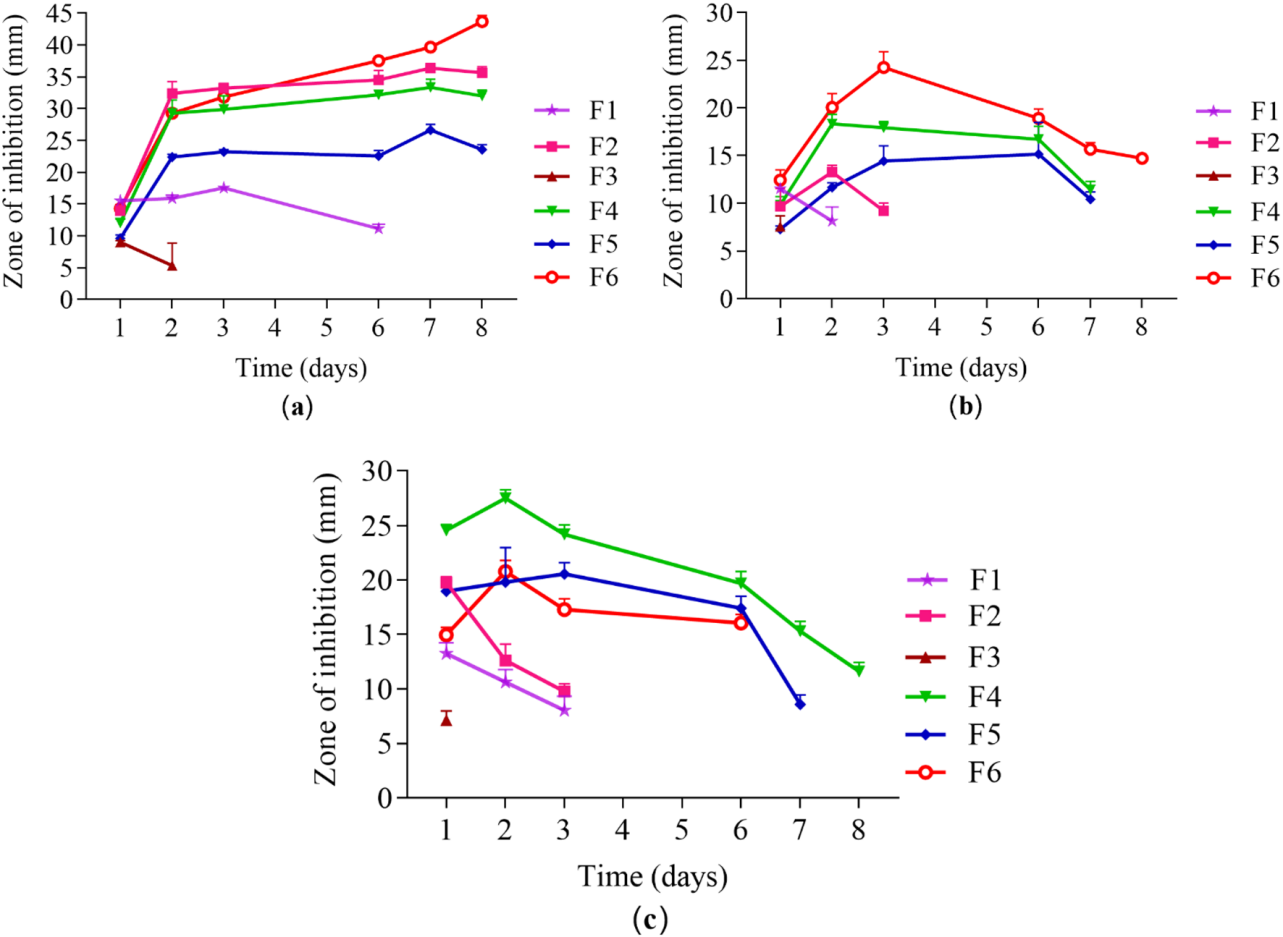
Persistence antimicrobial activity using diffusion method technique for wetted fabricated films against (**a**) *S. aureus*, (**b**) *E. coli*, and (**c**) *P. aeruginosa* measured in (mm), Where (F1): p-HEMA, (F2): RIF-loaded p-HEMA, (F3): CFX-loaded p-HEMA, (F4): (1:1) RIF:CFX-loaded p-HEMA, (F5): (1:3) RIF:CFX-loaded p-HEMA, (F6): (3:1) RIF:CFX-loaded p-HEMA. Data are represented as mean ± SD, (*n* = 6).

As observed in Fig. 6, fabricated films showed significant differences in antimicrobial persistence on successive days against *Staphylococcus aureus* (*S. aureus*), *Escherichia coli* (*E. coli*), and *Pseudomonas aeruginosa* (*P. aeruginosa*). Antimicrobial persistence is influenced by the amount of drugs ratios used over sequential transfers. The maximum antimicrobial persistence against *S. aureus* and *E. coli* was associated with (3:1) RIF: CFX-loaded p-HEMA discs (F6), with persistence lasting 8 days. While against *P. aeruginosa* was (1:1) RIF: CFX-loaded p-HEMA (F4), with persistence lasting 8 days. Antimicrobial persistence is influenced by the amount of drugs ratios used over sequential transfers. The maximum antimicrobial persistence against *S. aureus* and *E. coli* was observed with a higher load of RIF; (3:1) RIF: CFX-loaded p-HEMA discs (F6), which endured for 8 days. While the highest resistance to *P. aeruginosa* growth was illustrated when using equal ratios of the used microbicides (1:1) RIF: CFX-loaded p-HEMA (F4), with persistence continued to 8 days.

### Determination of biofilm-formation ability

**Figure 7:**
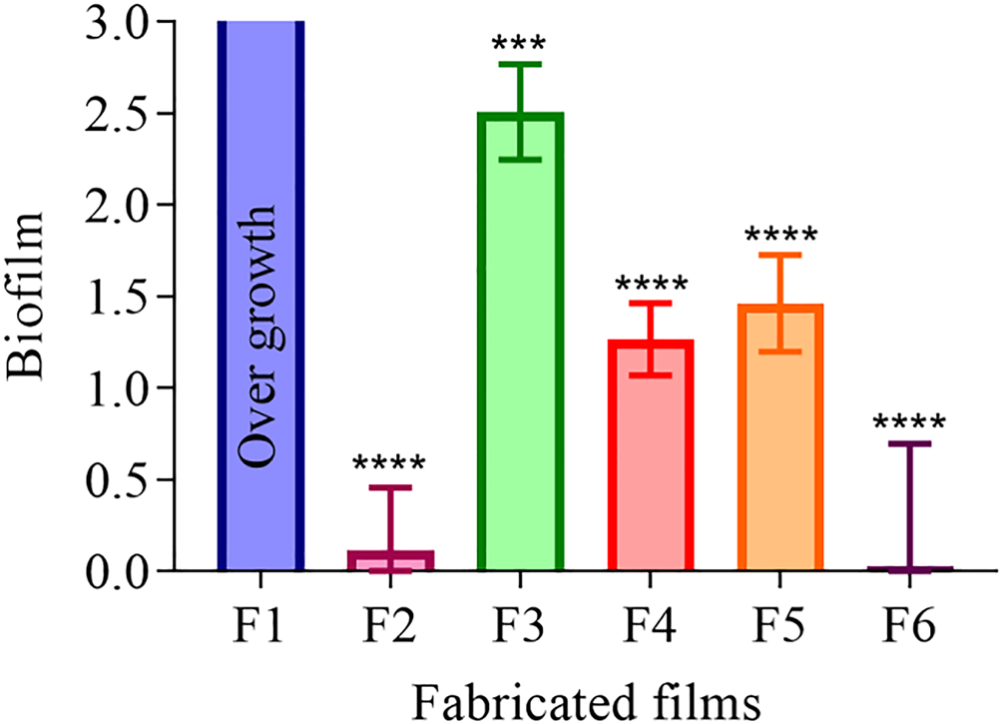
Mixed biofilm-formation ability of *S. aureus* ATCC 6538, *E. coli* ATCC 8739, and *P. aeruginosa* ATCC 9027 on the fabricated films surface. Where (F1): p-HEMA, (F2): RIF-loaded p-HEMA, (F3): CFX-loaded p-HEMA, (F4): (1:1) RIF:CFX-loadedp-HEMA, (F5): (1:3) RIF:CFX-loadedp-HEMA, and (F6): (3:1) RIF:CFX-loadedp-HEMA. Data are presented as mean ± SD, (*n* = 6). ***p-value < 0.001 significant difference between fabricated films.

The determination of viable count of microorganisms after treatment with MTT assay demonstrated microbial overgrowth on the surface of p-HEMA (F1). However, the addition of 0.5% (*w/w*) of RIF into p-HEMA (F2) illustrated lowering in microbial growth to 0.12 ± 0.34 (****p-value < 0.0001). Mixed biofilm-formation ability was 2.51 ± 0.26 (****p-value = 0.0001) on CFX-loaded p-HEMA (F3) surface. Also, on (1:1) RIF: CFX-loading p-HEMA (F4), and (1:3) RIF: CFX-loading p-HEMA (F5) surface mixed biofilm was 1.27 ± 0.2 and 1.46 ± 0.26 (****p-value < 0.0001) respectively. Moreover, it was found that the bacterial adherence was lowered in fabricated films containing a higher ratio of RIF as in (F6), it was 0.027 ± 0.67 (****p-value < 0.0001), as presented in Fig. 7.

## Discussion

The recent advancement in biomaterial sciences and their versatile employment in all medical fields changed the typical medical guidelines and traditional approaches in providing health care^40,41^. Developing medical devices is undeniably a direct application of those advancements in the aforementioned fields. However, the well-documented problem of developing sensitivity and microbial infections limited the use of those medical devices. For instance, the urinary medical devices (that include and are not limited to urinary catheters and stents) were reported to develop a microbial infection in 100% of all patients after catheterization within 1 month^42^. The only solution up to date is to replace the urinary device which will put the burden on the patient, medical staff, and cause cost-related sub-sequences^6^.

The main challenge in developing a microbial infection is the formation of biofilm, which is a complex structure that requires a higher concentration of microbicide up to 500 times to eradicate the microorganism considering the drug difficulty in penetrating the complex structure of polysaccharide and glycocalyx^42^. Moreover, it was found that microorganisms involved in biofilm-formation are not single species, but multispecies which makes the management even harder^8^. To address the mixed biofilm problem, a documented method to employ p-HEMA hydrogel in urinary catheters was adapted here and the hydrogel was exposed to multispecies microorganisms to detect their ability to delay biofilm formation. RIF was utilized attributed to its amphoteric nature that may prevent bacterial colonization^43,44^. Moreover, CFX is prescribed routinely in UTI^32^. p-HEMA was formed as control, void of a drug. Also, it was loaded with either RIF or CFX or a combination of both in different ratios. The formation of mixed biofilm was detected. Biofilm-formation results showed that the addition of microbicides significantly decreased biofilm-formation compared to p-HEMA, indicating that the properties of p-HEMA surface alone are not adequate and the loading of the drugs is quite crucial in resisting biofilm-formation. Furthermore, the physicochemical properties of the drug may contribute to the suppression of biofilm formation. For instance, the observed reduction in fabricated films containing a high amount of RIF may be attributed to the zwitterionic nature of the microbicide. Zwitterionic compound contains cation and anion groups which makes it highly hydrophilic. The hydrophilicity enables the formation of a hydration layer on the surface and repel nonspecific protein deposition on the surface through steric repulsion which subsequently reduces the pre-dispositioning factor of biofilm formation^43,44^.

The results from the persistence of antimicrobial activity were in compliance with the results obtained from the biofilm adhesion properties where the zone of inhibition over sequential transfers was affected by the type and amount of drug used. Essential to microbicide assessment was the evaluation of toxicity. HEK 293 cells cytotoxicity was evaluated by using an MTT assay that is based on the conversion of water-soluble yellow dye to an insoluble purple formazan by the action of mitochondrial reductase^45^. All fabricated films showed viable HEK 293 cells count higher than 80% and were deemed safe^46^, except RIF-loaded p-HEMA (F2) which showed the viability of 79.19 ± 14.92% which could be relatively safe. The viability lowering in (F2) may be attributed to the zwitterionic nature of the drug that allows its adsorption on the tested cell. Thus, the interaction between the p-HEMA and the tested drugs did not show toxicity on HEK 293 cells. Moreover, the fabricated films showed good swelling ability, which would allow oxygen permeability. Oxygen molecules can penetrate easily through free water network in the hydrogel to the cells allowing cellular activity^47^. The employment of polymeric coating layer in urinary stents and catheters should consider their capacity to swell. That is water uptake will expand the size and mass of the layer which—if not controlled—may lead to blockage. Therefore, swelling was studied at 2 different pH(s) considering normal and infected urine (if the infection was caused by urease forming bacteria). The observed lowering in EWC% of RIF-loaded p-HEMA (F2) at pH 5 could be attributed to the chemical nature of RIF which acts as zwitterion at pH 5 hindering its interaction with water^48^. Moreover, because RIF is zwitterionic, it would act as anion at pH 9 leading to higher ionization and subsequent enhancement in water uptake. The observed elevation in EWC% at pH 9 in all tested films is due to the ionization of the hydroxyl group in p-HEMA that leads to enhance water uptake and entrapment^49,50^. The protonation of the primary alcohol groups in p-HEMA would result in ionic repulsion leading to expansion and increasing the probabilities of interaction with water. Furthermore, the significant variations in ECW% in the tested films were correlated with T_*g*_. T_*g*_ is the temperature at which change of materials from an amorphous glassy solid to a rubber-like liquid occurs^51^. T_*g*_ reflects the polymer ability to move freely that at lower T_*g*_ value, as seen in Fig. 2. Loading p-HEMA with ratios of RIF and CFX decreased the EWC% possibly due to the increase of the interaction between the drugs and the polymer. Where the increase of the interaction between the polymer chain and the drugs would decrease the void volume and therefore reduce polymer chain mobility^52^, this result is also supported by T_*g*_ Fig. 2. Another important parameter affected by T_*g*_ is the mechanical properties^53^. For example, U.T.S increases with increasing T_*g*_, that is higher T_*g*_ means less void volume, and the required force to cause the brake, therefore, should be higher. On the contrary, elongation% increases when the void volume increases (lower T_*g*_), that is the stretching ability and polymer chain movement elevated^54,55^. Young’s modulus of the highest elongation% among the fabricated films has the lowest U.T.S. therefore, loaded p-HEMA with combined ratios of RIF and CFX (F4, F5, F6) elevate U.T.S, Young’s modulus, and decrease elongation%.

## Materials and methods

Rifampicin (MW 822.95 amu) was purchased from TGI (Tokyo, Japan), cefixime trihydrate (MW 507.5 amu) was kindly donated by Dar Al Dawa Pharmaceuticals (Amman, Jordan). 2-Hydroxyethyl methacrylate (2-HEMA) (MW 130.14 amu), ethylene glycol di methacrylate (EGDMA) (MW 198.22 amu), 2.2-Azobis (2-methyl-propionitrile) (AIBN) (MW 164.21 amu) were obtained from Sigma-Aldrich (Munich, Germany). Bacteria were obtained from American Type Culture Collection Organization (Manassas, Virginia, USA); *Staphylococcus aureus* (*S. aureus*) ATCC 6538, *Escherichia coli* (*E. coli*) ATCC 8739, and *Pseudomonas aeruginosa* (*P. aeruginosa*) ATCC 9027. Brain Heart Infusion broth (BHI) was purchased from biolab (Budapest, Hangary). Müller Hinton agar and Nutrient Broth (NB) obtained from HIMEDIA (Mumbai, India). Human embryonic kidney (HEK) cell line from ATCC (USA).

### Preparation of p-HEMA films and loading them with RIF, CFX, and combined ratios

**Figure 8:**
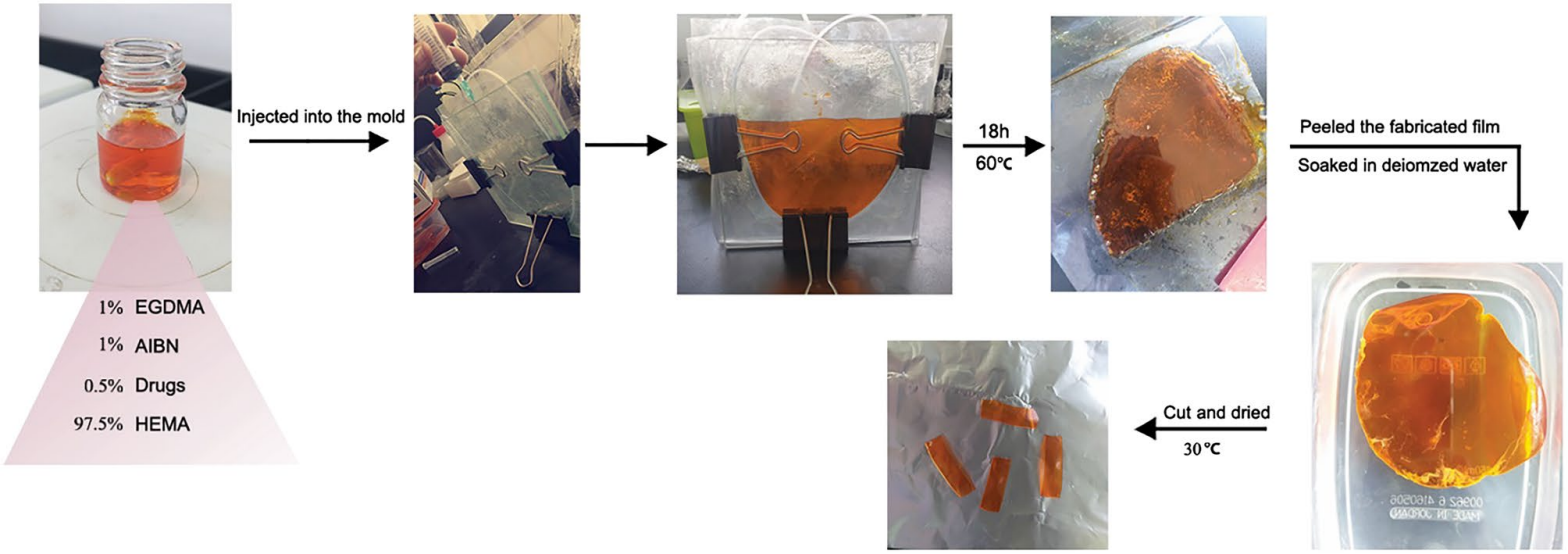
Films preparation steps include mixing the drug, polymer, cross linker and the initiator in a bottle, loading the mixtures in the molds, then soaking in distilled water after removal from oven. Finally, the resultant hydrogels are cut as required.

p-HEMA was prepared by the addition of a mixture of 1% (*w/w*) ethylene glycol-dimethacrylate (EGDMA) and 1% (*w/w*) of 2, 2′-azobisisobutyronitrile (AIBN) to a 98% (*w/w*) of 2-hydroxyethyl methacrylate (2-HEMA). After that, the mixture was continuously stirred for 10 min at 20 °C. Subsequently, the resulting mixture was injected slowly into the mold. The mold was in-house setup, containing two glass plates that were separated via a medical-grade silicone tube with a lumen diameter of 0.3 mm and wall thickness of 0.18 mm and clamped utilizing clips^56^. The polymerization reaction was carried out at 60 °C for 18 h. The fabricated film was peeled and soaked in deionized water to remove unreacted monomers, and this was confirmed by measuring the absorbance of acrylate till it reached zero. Acrylate absorbance was determined using a UV spectrophotometer (Shimadzu UV-1800 spectrometer (Kyoto, Japan)) at a maximum wavelength (λ_max_) of 220 nm. The soaked film was cut into the preferred shape, then dried to reach constant weight in an oven at 30 °C to produce xerogels. For drugs loading, the same procedure was carried out taking into account the amount of the drugs that will be included, 0.5% (*w/w*) of drugs was added to a mixture of 97.5% (*w/w*) 2-HEMA, 1% (*w/w*) EGDMA, and 1% (*w/w*) AIBN, as presented in Table 1. Film preparation is presented in Fig. 8.

**Table 1:** Formulations of the fabricated films, their components, and amount of ingredients.

Formulations	RIF (g)	CFX (g)	2-HEMA (g)	EGDMA (g)	AIBN (g)
F1 (control)	–	–	9.8	0.1	0.1
F2	0.05	–	9.75	0.1	0.1
F3	–	0.05	9.75	0.1	0.1
F4	0.025	0.025	9.75	0.1	0.1
F5	0.0125	0.0375	9.75	0.1	0.1
F6	0.0375	0.0125	9.75	0.1	0.1

### Thermal analysis using dynamic mechanical thermal analyser (DMTA)

The glass transition temperature (T_*g*_) of the dried films of p-HEMA and drugs-loaded p-HEMA was determined by utilizing Q800 DMTA (TA, New Castle, USA). The heating rate was 3 °C/min over a temperature range of 35 °C to 160 °C using tensile mode at an oscillatory frequency was 1 Hz. Dried films (*n* = 3) were cut into rectangular shapes with a dimension (30 mm length, 10 mm width, and 0.83 mm thickness using a digital caliper). The T_*g*_ values determined from the maximum peak of the tan δ, the dried films were fixed in the loading position inside the furnace^57,58^. Tan δ is represented as a dimensionless number, viewed as the mechanical damping factor represented as the ratio of loss and storage modulus, Eq. (1)^57^.

1 $$ {{\tan }}\delta = {\text{ Loss modulus}}/{\text{storage modulus}}. $$

### Mechanical analysis

Mechanical properties include U.T.S, elongation%, and Young’s modulus of the dried hydrogels were performed by using a TA XT plus Texture Analyzer (Stable Micro Systems)^59^. Dried films (30 mm length, 10 mm width, and 0.83 mm, *n* = 6) were clamped between the static lower grip and moveable upper grip, assuring that the length of the films below stress was equal to 20 mm. The upper clamp was raised at a constant rate (0.5 mm/s) until the fracture of the films happened. According to the stress–strain plot, the U.T.S, elongation% at the breakpoint, and Young’s modulus were calculated^54,55^.

### Equilibrium water content (swelling)

Films were cut into (30 mm length and 10 mm width, *n* = 6), then dried to reach constant weight in an oven at 30 °C. The dried films were carried into McCarthy bottles, each containing 15 mL of universal buffer pH 5 and pH 9 representing normal and infected urine with urease forming bacteria respectively at 20 °C. After 24 h, the soaked films were removed, blotted with filter paper to remove excess surface water, and reweighed. The equilibrium water content percent (EWC%) which is the percentage weight of water in the swollen polymer was calculated according to Eq. (2)^60^.

2 $$ {\text{EWC}}\% \, = \, \left( {{\text{M}}_{{\text{f}}} - \left( {{\text{M}}_{{\text{i}}} /{\text{M}}_{{\text{i}}} } \right)} \right) \, \times {1}00. $$

M_i_ and M_f_ represent the initial dried film mass and final film mass after buffer absorption, respectively^23^.

### Cytotoxic evaluation of the fabricated hydrogel using (MTT assay)

MTT (3-(4, 5-dimethylthiazol-2-yl)-2, 5-diphenyltetrazolium bromide) tetrazolium assay was applied to determine the cell proliferation, viability, and the potential cytotoxicity of fabricated films by MTT Cell Proliferation Assay (ATCC^®^ 30–1010 K)^61^. HEK 293 cells were seeded in a 96-well flat-bottom microtiter plate containing 100 μL medium at a density of 1 × 10^4^ cells/well and allowed to adhere at 37 °C in a humidified 5% CO_2_ incubator for 24 h. The dried films (discs, *n* = 6 in each case, with an average diameter of 3.735 ± 0.46 mm and average thickness of 0.83 ± 0.04 mm) sterilized using UV (FLUFRANCE, France) for 15 min and then soaked in RPMI 1640 media for softening before transferring to the wells and they were incubated for 24 h. Subsequently, 10 μL of MTT working solution was added to each well, and the plate placed in an incubator for 4 h. Then 100 μL Detergent Reagent (Stop Solution) was added to each well and the plate was placed for 2 h. Finally, the intensity of the formazan crystals (purple color) was determined using the Synergy™ HTX Multi-Mode Microplate Reader at 570 nm (BioTek, Winooski, VT, USA). Thus, %cell viability was calculated as shown in the Eq. (3), utilizing GraphPad Prism.

3 $$\text{Cell viability\% }= \frac{ (\text{Mean absorbance of treated cells}-\text{blank absorbance})}{\text{Mean absorbance of control cells }}\times 100.$$

### Persistence of antimicrobial activity

The persistence of antimicrobial activity of p-HEMA, and p-HEMA loaded with RIF, CFX, and combined ratios was investigated using three different bacterial strains *S. aureus* ATCC 6538, *E. coli* ATCC 8739, and *P. aeruginosa* ATCC 9027. Bacteria were incubated at 37 °C for 18 h in an Erlenmeyer flask including Nutrient Broth (NB). After 18 h bacteria were harvested by centrifugation and suspended in a new broth, the optical density (OD 540 nm) was evaluated to be equivalent to 1 × 10^8^ CFU/mL. The zone of inhibition (ZOI) was performed using the Kirby–Bauer disc diffusion technique with few modifications^62^, using wetted films (discs, *n* = 6 in each case, the average diameter of 5.53 ± 0.04 mm and the average thickness of 1.32 ± 0.01 mm). The wetted discs were fixed using sterile forceps on a Mueller Hinton agar (MHA) plate swabbed by the microbe. The plate was incubated for 24 h at 37 °C. After 24 h the zone of inhibition was measured in mm. The persistent antimicrobial activity of these antimicrobial agents was determined by transferring the discs to a freshly seeded plate every 24 h for 8 days^54^.

### Determination of biofilm-formation ability

Mixed biofilm-formation was investigated using three bacterial strains *S. aureus* ATCC 6538, *E. coli* ATCC 8739, and *P. aeruginosa* ATCC 9027. Mixed biofilm-formation ability was performed on dried hydrogel films of p-HEMA and drugs-loaded p-HEMA. The dried films (discs, *n* = 6 in each case, with an average diameter of 3.74 ± 0.46 mm and an average thickness of 0.83 ± 0.04 mm) were sterilized using a UV lamp (FLUFRANCE, France) for 15 min and placed into 96 well plates. Bacteria were incubated at 37 °C for 18 h in an Erlenmeyer flask including NB. After 18 h bacteria were harvested and suspended in Brain Heart Infusion broth (BHI). Bacteria were diluted with BHI, and the optical density (OD 540 nm) was evaluated to be equivalent to 1 × 10^6^ CFU/mL. Latter equal volumes of bacterial culture were mixed, and the total (OD 540 nm) was equivalent to 1 × 10^6^ CFU/mL. A 100 µL inoculum was transferred to the placed films on a 96-well plate. The plate was incubated at 37 °C for 90 min. After an adhesion period, non-attached bacteria were removed and fresh BHI was added. The plate was incubated at 37 °C for 24 h without shaking^52,63^. The films were washed with sterile phosphate buffer saline (PBS) to remove the loosely attached bacteria and were incubated for 3 h with 150 µL MTT solution. Then 100 μL Detergent Reagent was added to each well and the plate was placed for 15 min. The resulting absorbance OD was reading at 570 nm using the Synergy™ HTX Multi-Mode Microplate Reader (BioTek, Winooski, VT, USA)^64^.

### Statistical analysis

Data analysis was conducted using GraphPad Prism software version 7. The difference between groups was determined by one-way analysis of variance and two-way analysis (ANOVA) followed by the Tukey test. Data were represented as mean ± standard deviation (SD) and (*p*-value < 0.05) was deemed statistically significant.

## Conclusion

This study describes the development of urological biomaterials to minimize microbial growth and therefore reduce the incidence of CAUTIs. The fabricated films were synthesized from p-HEMA successfully and loaded with RIF, CFX, and combined ratios of both. Drug loaded hydrogel showed better persistence to microbial growth and biofilm formation. The physicochemical nature of the drugs had impact on the films. Where loading p-HEMA with RIF alone or RIF in highest ratio led to lower biofilm formation. Results show the ability of employing the p-HEMA with the loaded drugs in urinary biomaterials to increase their lifespan and subsequently patient acceptance.

## References

[1] Khan HA, Baig FK, Mehboob R (2017). Nosocomial infection: Epidemiology, prevention, control and surveillance. Asian Pac. J. Trop. Biomed..

[2] Gordon LB, Waxman MJ, Ragsdale L, Mermel LA (2013). Women presenting to the emergency department. J. Am. Geriatr. Soc..

[3] Tullus K (2019). Defining urinary tract infection by bacterial colony counts: A case for less than 100,000 colonies/mL as the threshold. Pediatr. Nephrol..

[4] Pelling H (2019). Bacterial biofilm formation on indwelling urethral catheters. Appl. Microbiol..

[5] Sabir N (2017). Causative pathogens and antibiotic resistance. Am. J. Infect. Control.

[6] Berne C, Ellison CK, Ducret A, Brun YV (2018). Bacterial adhesion at the single-cell level. Nat. Rev. Microbiol..

[7] Banerjee L, Pangule RC, Kane RS (2011). Antifouling coatings: Recent developments in the design of surfaces that prevent fouling by proteins, bacteria, and marine organisms. J. Adv. Mater..

[8] Sun PP (2018). Disintegration of simulated drinking water biofilms with arrays of microchannel plasma jets. NPJ Biofilms Microb..

[9] Huwaitat R, Sophie MC, Simon LP, Sreekanth P, Garry L (2021). Antibacterial and antibiofilm efficacy of synthetic polymyxin-mimetic lipopeptides. J. Pept. Sci..

[10] Ayyash M, Shehabi AA, Mahmoud NN, Al-Bakri AG (2019). Antibiofilm properties of triclosan with EDTA or cranberry as Foley Catheter lock solutions. J. Appl. Microbiol..

[11] Fan Y, Huang X, Chen J, Han B (2020). Formation of a mixed-species biofilm is a survival strategy for unculturable lactic acid bacteria and *Saccharomyces cerevisiae* in Daqu, a Chinese traditional fermentation starter. Front. Microbiol..

[12] Trifilio S (2015). Polymicrobial bacterial or fungal infections: Incidence, spectrum of infection, risk factors, and clinical outcomes from a large hematopoietic stem cell transplant center. Transplant. Infect. Dis..

[13] Jorge LS (2018). Outcomes and risk factors for polymicrobial posttraumatic osteomyelitis. J. Bone Jt. Infect..

[14] Bridier A, Sanchez-Vizuete P, Guilbaud M, Piard J, Naïtali M (2015). Biofilm-associated persistence of food-borne pathogens. Food Microbiol..

[15] Folbert EC (2017). Complications during hospitalization and risk factors in elderly patients with hip fracture following integrated orthogeriatric treatment. Arch. Orthop. Trauma Surg..

[16] Gordon D, Katlic M (2017). Chronic Catheter Associated Complications and Catheter-Associated Urinary Tract Infection.

[17] Ahmed S, Zugail AS, Pinar U, Irani J (2019). Evaluation of pain and catheter-related bladder discomfort relative to balloon volumes of indwelling urinary catheters: A prospective study. Investig. Clin. Urol..

[18] Dellimore KH, Helyer AR, Franklin SE (2013). A scoping review of important urinary catheter induced complications. J. Mater. Sci. Mater. Med..

[19] Hamill TM, Gilmore BF, Jones DS, Gorman SP (2014). Strategies for the development of the urinary catheter. Expert Rev. Med. Devices.

[20] Singhaa P, Locklin J, Handaa H (2016). A review of the recent advances in antimicrobial coatings for urinary catheters. Acta Biomater..

[21] Cardenas DD (2011). Intermittent catheterization with a hydrophilic-coated catheter delays urinary tract infections in acute spinal cord injury: A prospective, randomized, multicenter trial. PM&R.

[22] McCoyC P (2016). An infection-responsive approach to reduce bacterial adhesion in urinary biomaterials. Mol. Pharm..

[23] Francolini I, Vuotto C, Piozzi A, Donelli G (2017). Antifouling and antimicrobial biomaterials: An overview. APMIS.

[24] Ahmadabadi HY, Yu K, Kizhakkedathu JN (2020). Surface modification approaches for prevention of implant associated infections. Colloids Surf..

[25] Leuck AM (2015). Safety and efficacy of a novel silver-impregnated urinary catheter system for preventing catheter-associated bacteriuria: A pilot randomized clinical trial. Am. J. Infect. Control..

[26] Andersen MJ, Flores-Mireles AL (2020). Urinary catheter coating modifications: The Race against catheter-associated infections. Coating.

[27] Fisher LE (2015). Biomaterial modification of urinary catheters with antimicrobials to give long-term broadspectrum antibiofilm activity. J. Control. Release.

[28] Yang L, Whiteside S, Cadieux PA, Denstedt JD (2015). Ureteral stent technology: Drug-eluting stents and stent coatings. Asian J. Urol..

[29] Desai DG, Liao KS, Cevallos ME, Trautner BW (2010). Silver or nitrofurazone impregnation of urinary catheters has a minimal effect on uropathogen adherence. JURO.

[30] Timothy TR (2012). Antimicrobial effects of nanofiber poly(caprolactone) tissue scaffolds releasing rifampicin. J. Mater. Sci. Mater. Med..

[31] Angiolini L, Agnes M, Cohen B, Yannakopoulou K, Douhal A (2017). Formation; characterization and PH dependence of rifampicin:heptakis(2,6-di-o-methyl)-β-cyclodextrin complexes. Int. J. Pharm..

[32] Shooshtari NM, Ghazi MM (2017). An investigation of the photocatalytic activity of nano α-Fe_2_O_3_/ZnO on the photodegradation of CFXixime trihydrate. Chem. Eng. Sci..

[33] Huh KM, Kang HC, Lee YJ, Bae YH (2012). PH-sensitive polymers for drug delivery. Macromol. Res..

[34] Trzebicka B (2019). Thermoresponsive P(HEMA-co-OEGMA) copolymers: Synthesis, characteristics and solution behavior. RSC.

[35] Sari SMC, Benmouna M, Mahlous M, Kaci M (2013). Swelling behavior of poly (2-hydroxyethyl methacrylate) copolymer gels. MATEC Web Conf..

[36] Sun DD, Rob JuTC, Lee PI (2012). Enhanced kinetic solubility profiles of indomethacin amorphous solid dispersions in poly(2-hydroxyethyl methacrylate) hydrogels. Eur. J. Pharm. Biopharm..

[37] Passos MF (2016). pHEMA hydrogels. J. Therm. Anal. Calorim..

[38] Omidian H, Park K, Kandalam U, Rocca JG (2010). Swelling and mechanical properties of modified HEMA-based superporous hydrogels. J. Bioact. Compat. Polym..

[39] Rapado M, Peniche C (2015). Synthesis and characterization of pH and temperature responsive poly(2-hydroxyethyl methacrylate-co-acrylamide) hydrogels. Polimeros.

[40] Tominaga GJ (2014). Eliminating catheter-associated urinary tract infections in the intensive care unit: Is it an attainable goal?. Am. J. Surg..

[41] Stickler D (1999). Biofilms. Curr. Opin. Microbiol..

[42] Zhu Z, Wang Z, Li S, Yuan X (2018). Antimicrobial strategies for urinary catheters. J. Biomed. Mater. Res..

[43] Phuyal A, Ojha PK, Guragain B, Chaudhary NK (2019). Phytochemical screening, metal concentration determination, antioxidant activity, and antibacterial evaluation of *Drymaria diandra* plant. J. Basic Appl..

[44] Hussain A, Altamimi M, Alshehri S, SarimImam S, Singh SK (2020). Vesicular elastic liposomes for transdermal delivery of rifampicin: In-vitro, in-vivo and in silico GastroPlus™ prediction studies. Eur. J. Pharm. Sci..

[45] Laothumthut T, Jantarat J, Paemanee A, Roytrakul S, Chunhabundit P (2015). shotgun proteomics analysis of proliferating STRO-1-positive human dental pulp cell after exposure to a nacreous water-soluble matrix. Clin. Oral Investig..

[46] López-García J, Lehocký M, Humpolíček P, Sáha P (2014). HaCaT keratinocytes response on antimicrobial atelocollagen substrates: Extent of cytotoxicity, cell viability and proliferation. J. Funct. Biomater..

[47] Emter R, Natsch A (2015). A fast resazurin-based live viability assay is equivalent to the MTT-test in the keratinosens assay. Toxicol. In Vitro.

[48] Tan Y, Leonhard M, Schneider-stickler B (2017). Evaluation of culture conditions for mixed bio Fi Lm formation with clinically isolated non-albicans candida species and *Staphylococcus epidermidis* on silicone. Microb. Pathog..

[49] Wicher B, Pyta K, Przybylski P, Gdaniec M (2017). Solvates of zwitterionic rifampicin: Recurring packing motifs via nonspecific interactions. Cryst. Growth Des..

[50] Desseaux S (2016). Swelling behavior and nanomechanical properties of (peptide-modified) poly(2-hydroxyethyl methacrylate) and poly(poly(ethylene glycol) methacrylate) brushes. Macromolecules.

[51] Pandi P, Bulusu R, Kommineni N, Khan W, Singh M (2020). Amorphous solid dispersions: An update for preparation, characterization, mechanism on bioavailability, stability, regulatory considerations. Int. J. Pharm..

[52] Tarawneh O, Alwahsh W, Abul-Futouh H, Al-Samad L, Hamadneh L, Abu Mahfouz H, Fadhil Abed A (2021). Determination of antimicrobial and antibiofilm activity of combined LVX and AMP impregenated in p(HEMA) hydrogel. Appl. Sci..

[53] Wang YH, Wang WH, Zhang Z, Xu L, Li P (2016). Study of the glass transition temperature and the mechanical properties of PET/modified silica nanocomposite by molecular dynamics simulation. Eur. Polym..

[54] Jones DS, Mccoy CP, Andrews GP, Mccrory RM, Gorman SP (2015). Hydrogel antimicrobial capture coatings for endotracheal tubes: A pharmaceutical strategy designed to prevent ventilator-associated pneumonia. Mol. Pharm..

[55] Raigond P (2019). Antimicrobial activity of potato starch-based active biodegradable nanocomposite films. Potato Res..

[56] Irwin NJ, McCoy CP, Jones DS, Gorman SP (2013). Infection-responsive drug delivery from urinary biomaterials controlled by a novel kinetic and thermodynamic approach. Pharm. Res..

[57] Saba N, Jawaid M, Alothman O, Paridah MT (2016). A review on dynamic mechanical properties of natural fibre reinforced polymer composites. Constr. Build. Mater..

[58] Tarawneh O, Hamadneh I, Huwaitat R, Al-Assi AR, El Madani A (2021). Characterization of chlorhexidine-impregnated cellulose-based hydrogel films intended for the treatment of periodontitis. Biomed. Res. Int..

[59] Jiang D, Smith DE (2017). Anisotropic mechanical properties of oriented carbon fiber filled polymer composites produced with fused filament fabrication. Addit. Manuf..

[60] Pradhan AK, Rana PK, Sahoo PK (2015). Biodegradability and swelling capacity of kaolin based chitosan-g-PHEMA nanocomposite hydrogel. Int. J. Biol. Macromol..

[61] Van Tonder A, Joubert AM, Cromarty AD (2015). Limitations of the 3-(4,5-dimylthiazol-2-Yl)-2,5-diphenyl-2H-tetrazolium bromide (MTT) assay when compared to three commonly used cell enumeration assays. BMC Res. Notes.

[62] Ashish P, Ojha PK, Guragain B, Chaudhary NK (2019). Phytochemical screening, metal concentration determination, antioxidant activity, and antibacterial evaluation of *Drymaria diandra* plant. J. Basic Appl..

[63] Mourad R, Helaly F, Darwesh O, El-Sawy S (2019). Antimicrobial and physicomechanical natures of silver nanoparticles incorporated into silicone-hydrogel films. Cont. Lens Anterior Eye.

[64] Crouzet-Bertrand X, Venier AG, Badoz M, Husson C, Talon D (2007). Control of the duration of urinary catheterization: Impact on catheter-associated urinary tract infection. J. Hosp. Infect..

